# Frailty index and tolerability of SGLT2 inhibitors in elderly (≥75 years) patients with heart failure

**DOI:** 10.1097/MD.0000000000049184

**Published:** 2026-07-10

**Authors:** Zimeng Li, Wei Deng

**Affiliations:** a The First Clinical College of Wuhan University, Wuhan, Hubei Province, China; b Department of Heart Failure and Cardiomyopathy, Renmin Hospital of Wuhan University, Wuhan, Hubei Province, China.

**Keywords:** elderly patients, frailty, frailty index/fried frailty phenotype, heart failure, SGLT2 inhibitors, tolerability

## Abstract

Sodium–glucose cotransporter 2 inhibitors (SGLT2i) are cornerstone therapies for heart failure (HF), but data on tolerability in frail elderly patients (≥75 years) remain limited. This study investigates the relationship between frailty status and SGLT2i tolerability in elderly HF patients and its impact on short-term clinical outcomes. We retrospectively enrolled 115 patients aged ≥75 years with HF who received SGLT2i between February 2023 and February 2024. Patients were stratified by Fried frailty phenotype score into a non-frail group (score < 3, n = 64) and a frail group (score ≥ 3, n = 51). The primary outcome was a 6-month composite of SGLT2i intolerance (permanent discontinuation, dose reduction, or interruption ≥ 7 days). Secondary outcomes included early intolerance (≤30 days), adverse events, and clinical outcomes. Between-group comparisons were performed using the *t* test, Mann–Whitney *U* test, or χ^2^ test as appropriate. Multivariable logistic regression was used to assess the association between frailty and intolerance, with results presented as adjusted odds ratios (OR) with 95% confidence intervals (CI). Cox regression was applied for time-to-event outcomes, reported as hazard ratios (HR) with 95% CI. Over 6 months, 32 patients (27.8%) experienced SGLT2i intolerance. The frail group had a significantly higher intolerance rate than the non-frail group (39.2% vs 18.8%; adjusted OR = 2.31, 95% CI: 1.06–5.04, *P* = .035). Early intolerance (≤30 days) was also more frequent in frail patients (23.5% vs 9.4%; adjusted OR = 2.89, 95% CI: 1.08–7.76, *P* = .034). Adverse events such as symptomatic hypotension/volume depletion, AKI, and infections were more common in frail patients. Clinical outcomes including HF-related rehospitalization (29.4% vs 15.6%), all-cause rehospitalization (37.3% vs 21.9%), and all-cause mortality (17.6% vs 7.8%) were higher in frail patients, though differences were not statistically significant. The association between frailty and intolerance was more pronounced in patients with eGFR < 45 mL/min/1.73m^2^ (interaction *P* = .041). In elderly (≥75 years) HF patients, frailty is significantly associated with increased SGLT2i intolerance, particularly in those with reduced renal function, highlighting the need for individualized treatment strategies.

## 1. Introduction

Heart failure (HF) is a major public health issue worldwide and is particularly prevalent among the elderly. With the acceleration of population aging, both the incidence and prevalence of HF have been rising rapidly.^[1,2]^ Epidemiological data show that individuals aged ≥75 years now account for more than half of all HF cases. Despite advances in pharmacological and device-based therapies in recent years, rehospitalization, disability, and mortality rates remain high among older patients with HF.^[3,4]^ The main reasons include frequent comorbidities, polypharmacy, and reduced renal and hepatic functional reserves, which predispose this population to drug-related adverse events and increase the complexity of treatment.^[5]^

Frailty is a multidimensional geriatric syndrome characterized by decreased physiological reserve and heightened vulnerability to external stressors, and it is highly prevalent among older HF patients.^[6]^ The fried frailty phenotype (FFP), consisting of 5 domains – unintentional weight loss, exhaustion, reduced grip strength, slower gait speed, and decreased physical activity – is currently the most widely used clinical assessment tool for frailty.^[6,7]^ Previous studies have shown that approximately 30% to 50% of HF patients aged ≥70 years exhibit varying degrees of frailty. Frailty has been associated not only with higher risks of rehospitalization, poorer quality of life, and increased mortality, but may also influence drug adherence and tolerability, making it an important factor in clinical decision-making.^[8,9]^

Sodium–glucose cotransporter 2 inhibitors (SGLT2i), such as dapagliflozin and empagliflozin, have recently emerged as a cornerstone in HF therapy.^[10]^ Large randomized controlled trials (e.g., DAPA-HF, EMPEROR-Preserved) have demonstrated the significant benefits of SGLT2i in reducing HF hospitalization and improving prognosis, regardless of diabetes status.^[11]^ However, the mean age of participants in these pivotal trials was around 65 to 68 years, with relatively few patients aged ≥75 years, particularly those with frailty, thereby limiting the generalizability of the results to this high-risk subgroup.^[12]^ Consequently, current evidence is insufficient to determine whether frail older patients with HF can tolerate SGLT2i therapy well.

In clinical practice, physicians are often cautious in prescribing SGLT2i to frail elderly patients due to concerns about adverse events such as hypotension, volume depletion, acute kidney injury (AKI), electrolyte disturbances, or genitourinary infections.^[13]^ Frail patients generally present with compromised hemodynamic stability, reduced muscle mass, poor nutritional status, and a high burden of comorbidities, all of which may increase the risk of drug-related adverse events.^[13]^ Although some studies have explored the safety of SGLT2i in older HF populations, systematic investigations specifically addressing the relationship between frailty and SGLT2i tolerability remain scarce.

Clarifying the relationship between frailty and SGLT2i tolerability has important clinical implications. On one hand, reducing treatment intensity due to tolerability concerns may lead to insufficient therapeutic benefit; on the other, disregarding frailty status and intensifying therapy may increase the risk of adverse outcomes. Therefore, identifying the impact of frailty on SGLT2i tolerability could help optimize individualized treatment strategies and improve the safety–benefit profile in this population.

Based on this rationale, the present study employed a single-center retrospective cohort design, enrolling patients aged ≥75 years with HF treated at our institution between February 2023 and February 2024. Frailty status was assessed using the fried frailty phenotype, and the association between frailty and SGLT2i intolerance was systematically analyzed. In addition, we examined early intolerance events within 30 days, the spectrum of adverse events, and prognosis-related clinical outcomes (HF rehospitalization, all-cause rehospitalization, and mortality). The findings aim to provide real-world evidence to guide the rational application of SGLT2i in frail elderly patients with HF.

## 2. Materials and methods

### 2.1. Study population

This study was approved by the Ethics Committee of Renmin Hospital of Wuhan University. This was a single-center retrospective cohort study. Patients were consecutively enrolled from among those with heart failure aged ≥75 years who were admitted to or followed up at our hospital between February 2023 and February 2024. According to the fried frailty phenotype (FFP) assessed during a clinically stable period, patients were divided into 2 groups: the non-frail group (n = 64, FFP score < 3, including robust and pre-frail) and the frail group (n = 51, FFP score ≥ 3). All enrolled patients had exposure to sodium-glucose cotransporter 2 inhibitors (SGLT2i), predominantly dapagliflozin or empagliflozin, for the evaluation of tolerability (defined as drug discontinuation, dose reduction, interruption ≥ 7 days, or relevant adverse events).

### 2.2. Inclusion and exclusion criteria

Inclusion criteria: age ≥ 75 years; hospitalized or attending outpatient clinics at our hospital between February 01, 2023 and February 29, 2024 with a confirmed diagnosis of heart failure (HF), based on physician records and/or imaging and laboratory findings (e.g., echocardiographic left ventricular ejection fraction [LVEF], B-type natriuretic peptide [BNP]/N-terminal pro-B-type natriuretic peptide [NT-proBNP], typical clinical signs, or discharge diagnosis); initiation of SGLT2i (dapagliflozin or empagliflozin) at our hospital during the study period, or continuation of therapy with baseline status confirmed at the time of first prescription renewal; completion of fried frailty phenotype (FFP) assessment during a relatively stable phase (24–72 hours prior to discharge for inpatients, or within 7 days after prescription for outpatients); complete baseline data available, including demographics, vital signs (systolic blood pressure/orthostatic blood pressure), renal function (serum creatinine [Scr] and estimated glomerular filtration rate [eGFR]), concomitant medications (diuretics, renin-angiotensin-aldosterone system [RAAS] inhibitors, β-blockers, etc) and medical history; at least 4 out of 5 FFP items evaluable; and at least 1 follow-up record (outpatient, inpatient, prescription renewal, telephone follow-up, or electronic medical record) that could be used to determine whether intolerance events occurred.

Exclusion criteria: prior use of SGLT2i with prescription records within 90 days before the index date (defined as the date of the first prescription of SGLT2i at our hospital or the initiation of therapy during hospitalization), to avoid bias from including “previously tolerant” patients; severely impaired renal function, including chronic dialysis, eGFR < 15 mL/min/1.73m^2^, or a history of kidney transplantation; baseline admission for cardiogenic shock or requiring hemodynamic support (vasopressors, IABP [intra-aortic balloon pump]/ECMO [extracorporeal membrane oxygenation]) or mechanical ventilation, making it impossible to complete reliable FFP assessment during a stable phase; and absolute contraindications or high-risk conditions for SGLT2i therapy, including a history of diabetic ketoacidosis (including euglycemic) or active severe urinary/genital fungal infection requiring systemic therapy.

### 2.3. Frailty assessment

Frailty was assessed using the fried frailty phenotype (FFP) scale during a clinically stable phase. The scale consisted of 5 components: unintentional weight loss (>4.5 kg in the past year); self-reported exhaustion (from CES-D [Center for Epidemiologic Studies Depression Scale] items); reduced grip strength measured by dynamometer (below sex- and BMI-specific thresholds); slowed walking speed (4.5 m walking time, below sex- and height-specific thresholds); and low physical activity (based on the Minnesota Leisure Time Physical Activity Questionnaire). Each positive item was scored 1, with a total score ranging from 0 to 5. Patients were classified as non-frail (score 0), pre-frail (score 1–2), or frail (score ≥ 3). For the main analysis, non-frail and pre-frail patients were combined into the non-frail group (score < 3) and compared with the frail group (score ≥ 3).

### 2.4. Outcomes

#### 2.4.1. Primary outcomes

The primary endpoint (6-month composite intolerance endpoint) was defined as any event related to SGLT2i therapy that led to permanent discontinuation, dose reduction (if applicable), or interruption of therapy for ≥7 days.

#### 2.4.2. Secondary outcomes

Secondary outcomes included early intolerance within 30 days, the incidence of each specific adverse event (symptomatic hypotension/volume depletion, AKI [KDIGO definition], urinary or genital tract infection/fungal infection, electrolyte disturbances [such as hyponatremia], diabetic ketoacidosis [including euglycemic], severe hypoglycemia, etc), duration of therapy (time to discontinuation by KM/Cox analysis), as well as HF-related hospitalization, all-cause hospitalization, and all-cause death. All adverse events were independently assessed and adjudicated by 2 investigators.

### 2.5. Data collection

Data were collected through the hospital information system (HIS), laboratory information system (LIS), and structured telephone follow-up.

#### 2.5.1. Demographic data

Demographic data included age and sex.

#### 2.5.2. Anthropometric measures

Anthropometric measures included body mass index (BMI).

#### 2.5.3. Heart failure-related indicators

Heart failure-related indicators included cardiac function assessed by NYHA classification, cardiac structure and function assessed by echocardiographic LVEF, and biomarkers including NT-proBNP levels. Comorbidities included a history of hypertension, diabetes, coronary artery disease, atrial fibrillation, chronic kidney disease (CKD), and chronic obstructive pulmonary disease (COPD).

#### 2.5.4. Laboratory examinations

Laboratory examinations included renal function (serum creatinine [Scr] and eGFR), and nutritional and metabolic indicators such as serum albumin, hemoglobin, and serum sodium.

#### 2.5.5. Medication data

Medication data included baseline cardiovascular therapy, such as ACEI/ARB/ARNI, β-blockers, MRA, and loop diuretics (calculated as furosemide-equivalent dose). The type of SGLT2i (dapagliflozin or empagliflozin) was also recorded.

#### 2.5.6. Other geriatric syndromes

Other geriatric syndromes included history of falls within the past year, cognitive function assessed by MMSE or clinical records, nutritional status assessed by MNA-SF (≤11 indicating risk of malnutrition), depressive symptoms assessed by GDS-15 (≥5 suggesting possible depression), polypharmacy defined as the long-term use of ≥5 prescription drugs, and urinary incontinence confirmed by patient report or medical records.

### 2.6. Statistical analysis

All statistical analyses were performed using SPSS 26.0 software. Continuous variables were expressed as mean ± standard deviation or median (interquartile range), and comparisons between groups were conducted using an independent-samples *t* test or Mann–Whitney *U* test. Categorical variables were expressed as counts (%) and compared using the χ^2^ test or Fisher exact test. Missing data were minimal (<5% for all variables) and were handled by complete case analysis.

For the primary outcome of SGLT2i intolerance (binary composite endpoint at 6 months), given the absence of precise time-to-event data for intolerance events, multivariable logistic regression was used to evaluate the association between frailty status and SGLT2i intolerance, with adjusted odds ratios (OR) and 95% confidence intervals (CI) calculated. Covariates were selected based on baseline imbalances (*P* < .05) and clinical relevance, including age, BMI, eGFR, NYHA class, loop diuretic dose, and chronic kidney disease.

For time-to-event clinical outcomes (HF-related rehospitalization, all-cause rehospitalization, and all-cause mortality), for which the exact time of event occurrence was known, Cox proportional hazards regression models were used, with hazard ratios (HR) and 95% CI calculated. The proportional hazards assumption was tested using Schoenfeld residuals and was satisfied.

Subgroup analyses were performed with interaction terms between frailty and prespecified variables (eGFR, SBP, loop diuretic dose, HF phenotype, and diabetes status) entered into the regression models; interaction *P* values were calculated using likelihood ratio tests. Nagelkerke *R*^2^ was calculated for logistic regression models to assess model fit. All tests were 2-sided, and a *P* value < .05 was considered statistically significant.

## 3. Results

### 3.1. Comparison of baseline characteristics between the 2 groups

A total of 115 patients aged ≥75 years with heart failure were included in this study and divided into the non-frail group (n = 64) and the frail group (n = 51) according to the Fried score. As shown in Table 1, compared with the non-frail group, patients in the frail group were older (82.6 ± 4.5 years vs 80.1 ± 3.8 years, *P* = .012), had a lower BMI (22.7 ± 3.2 kg/m^2^ vs 24.1 ± 3.0 kg/m^2^, *P* = .048), and a higher proportion of NYHA class III to IV (64.7% vs 43.8%, *P* = .024). The frail group also had a higher prevalence of chronic kidney disease (52.9% vs 29.7%, *P* = .015), worse renal function (eGFR: 48.1 ± 14.0 vs 56.2 ± 13.8 mL/min/1.73 m^2^, *P* = .009), and poorer nutritional status (albumin: 35.7 ± 4.2 vs 37.8 ± 3.9 g/L, *P* = .022; hemoglobin: 114.5 ± 15.0 vs 121.5 ± 13.8 g/L, *P* = .018). In addition, the frail group received higher doses of loop diuretics (median 60 mg vs 40 mg, *P* = .031). No significant differences were observed between the 2 groups in other baseline characteristics.

**Table 1:** Baseline characteristics of elderly (≥75 years) heart failure patients (n = 115).

Variable	Overall (n = 115)	Non-frail (n = 64)	Frail (n = 51)	*P* value
Demographics				
Age, yr, mean ± SD	81.2 ± 4.2	80.1 ± 3.8	82.6 ± 4.5	.012
Male, n (%)	62 (53.9)	37 (57.8)	25 (49.0)	.356
BMI, kg/m^2^, mean ± SD	23.5 ± 3.1	24.1 ± 3.0	22.7 ± 3.2	.048
Heart failure assessment				
NYHA class III–IV, n (%)	61 (53.0)	28 (43.8)	33 (64.7)	.024
LVEF, %, mean ± SD	42.3 ± 9.6	43.8 ± 9.2	40.4 ± 10.0	.086
NT-proBNP, pg/mL, median (IQR)	3210 (1880–5180)	2890 (1760–4710)	3650 (2090–5620)	.118
Comorbidities				
Hypertension, n (%)	83 (72.2)	44 (68.8)	39 (76.5)	.363
Diabetes mellitus, n (%)	55 (47.8)	28 (43.8)	27 (52.9)	.348
Coronary artery disease, n (%)	41 (35.7)	20 (31.3)	21 (41.2)	.276
Atrial fibrillation, n (%)	39 (33.9)	18 (28.1)	21 (41.2)	.132
Chronic kidney disease, n (%)	46 (40.0)	19 (29.7)	27 (52.9)	.015
COPD, n (%)	22 (19.1)	11 (17.2)	11 (21.6)	.562
Laboratory tests				
Serum creatinine, mg/dL, mean ± SD	1.23 ± 0.41	1.15 ± 0.36	1.33 ± 0.45	.041
eGFR, mL/min/1.73m^2^, mean ± SD	52.6 ± 14.2	56.2 ± 13.8	48.1 ± 14.0	.009
Serum sodium, mmol/L, mean ± SD	138.6 ± 3.7	139.2 ± 3.4	137.8 ± 3.9	.078
Albumin, g/L, mean ± SD	36.9 ± 4.1	37.8 ± 3.9	35.7 ± 4.2	.022
Hemoglobin, g/L, mean ± SD	118.3 ± 14.6	121.5 ± 13.8	114.5 ± 15.0	.018
Medications				
ACEI/ARB/ARNI, n (%)	68 (59.1)	39 (60.9)	29 (56.9)	.675
β-blockers, n (%)	74 (64.3)	43 (67.2)	31 (60.8)	.492
MRA, n (%)	52 (45.2)	27 (42.2)	25 (49.0)	.463
Loop diuretics, n (%)	85 (73.9)	43 (67.2)	42 (82.4)	.072
Loop diuretic dose (furosemide equivalent, mg/day), median (IQR)	40 (20–80)	40 (20–60)	60 (40–80)	.031
SGLT2i agent: Dapagliflozin, n (%)	64 (55.7)	36 (56.3)	28 (54.9)	.884
SGLT2i agent: Empagliflozin, n (%)	51 (44.3)	28 (43.8)	23 (45.1)	.884

ACEI = angiotensin-converting enzyme inhibitor, ARB = angiotensin receptor blocker, ARNI = angiotensin receptor-neprilysin inhibitor, BMI = body mass index, COPD = chronic obstructive pulmonary disease, eGFR = estimated glomerular filtration rate, IQR = interquartile range, LVEF = left ventricular ejection fraction, MRA = mineralocorticoid receptor antagonist, NT-proBNP = N-terminal pro-B-type natriuretic peptide, NYHA = New York Heart Association, SD = standard deviation, SGLT2i = sodium-glucose cotransporter 2 inhibitor.

* Data are presented as mean ± standard deviation (SD), median (interquartile range, IQR), or number (%). Group comparisons were performed using independent-samples *t* test for normally distributed continuous variables, Mann–Whitney *U* test for non-normally distributed continuous variables, and χ^2^ test or Fisher exact test for categorical variables, as appropriate.

### 3.2. Frailty phenotype and geriatric syndrome assessment

The results of frailty components and other geriatric syndrome assessments in the 2 groups are shown in Table 2. All 5 components of the fried frailty phenotype (unintentional weight loss, exhaustion, reduced grip strength, slower walking speed, low physical activity) were significantly more prevalent in the frail group compared with the non-frail group (all *P* < .01). In addition, the frail group had higher rates of other geriatric syndromes, including a history of falls within the past year (29.4% vs 10.9%), cognitive impairment (25.5% vs 7.8%), risk of malnutrition (35.3% vs 12.5%), depression (27.5% vs 9.4%), polypharmacy (≥5 medications; 76.5% vs 50.0%), and urinary incontinence (35.3% vs 14.1%), all with statistically significant differences (all *P* < .05).

**Table 2:** Frailty components and other geriatric conditions in elderly (≥75 years) heart failure patients.

Variable	Overall (n = 115)	Non-frail (n = 64)	Frail (n = 51)	*P* value
Frailty components (fried phenotype)				
Unintentional weight loss, n (%)	29 (25.2)	9 (14.1)	20 (39.2)	.002
Exhaustion, n (%)	41 (35.7)	13 (20.3)	28 (54.9)	<.001
Weak grip strength, n (%)	56 (48.7)	18 (28.1)	38 (74.5)	<.001
Slow walking speed, n (%)	47 (40.9)	15 (23.4)	32 (62.7)	<.001
Low physical activity, n (%)	52 (45.2)	16 (25.0)	36 (70.6)	<.001
Other geriatric conditions				
History of falls (past 1 yr), n (%)	22 (19.1)	7 (10.9)	15 (29.4)	.014
Cognitive impairment, n (%)	18 (15.7)	5 (7.8)	13 (25.5)	.011
Malnutrition risk (MNA-SF ≤ 11), n (%)	26 (22.6)	8 (12.5)	18 (35.3)	.004
Depression (GDS ≥ 5), n (%)	20 (17.4)	6 (9.4)	14 (27.5)	.016
Polypharmacy (≥5 drugs), n (%)	71 (61.7)	32 (50.0)	39 (76.5)	.006
Urinary incontinence, n (%)	27 (23.5)	9 (14.1)	18 (35.3)	.009

GDS = Geriatric Depression Scale, MNA-SF = Mini Nutritional Assessment-Short Form.

### 3.3. SGLT2 inhibitor tolerability outcomes

The incidence of the primary composite endpoint of SGLT2i intolerance within 6 months is shown in Table 3. Overall, 32 patients (27.8%) experienced intolerance events. The incidence of intolerance was significantly higher in the frail group compared with the non-frail group (39.2% vs 18.8%). After multivariable adjustment, frailty remained independently associated with SGLT2i intolerance (adjusted OR = 2.31, 95% CI: 1.06–5.04, *P* = .035).

**Table 3:** Six-month SGLT2i intolerance (primary composite endpoint).

Group	n/N (%)	Risk difference, % (95% CI)	Unadjusted OR (95% CI)	Adjusted OR (95% CI)	*P* value
Non-frail	12/64 (18.8%)	–	1.00 (ref)	1.00 (ref)	–
Frail	20/51 (39.2%)	20.4 (6.3–34.5)	2.83 (1.24–6.47)	2.31 (1.06–5.04)	.035
Overall	32/115 (27.8%)	–	–	–	–

CI = confidence interval, OR = odds ratio, SGLT2i = sodium–glucose cotransporter 2 inhibitors.

* n/N (%) indicates the number of patients who experienced the intolerance event within each group divided by the total number of patients in that group, expressed as a percentage. Nagelkerke *R*^2^ for the multivariable logistic regression model was 0.187, reflecting the proportion of variance in the outcome explained by the included predictors.

### 3.4. Clinical outcome events

The incidence of 6-month clinical outcomes is shown in Table 4. The frail group had higher rates of HF rehospitalization (29.4% vs 15.6%), all-cause rehospitalization (37.3% vs 21.9%), and all-cause mortality (17.6% vs 7.8%) compared with the non-frail group. However, after multivariable adjustment using Cox regression, these differences did not reach statistical significance (adjusted HR: 1.92, 1.84, and 2.12, respectively; all *P* > .05).

**Table 4:** Six-month clinical outcomes (HF-related rehospitalization, all-cause rehospitalization, all-cause mortality).

Outcome	Non-frail n/N (%)	Frail n/N (%)	Risk difference, % (95% CI)	Adjusted HR (95% CI)	*P* value
HF-related rehospitalization	10/64 (15.6%)	15/51 (29.4%)	13.8 (0.8–26.8)	1.92 (0.87–4.23)	.105
All-cause rehospitalization	14/64 (21.9%)	19/51 (37.3%)	15.4 (1.0–29.8)	1.84 (0.91–3.73)	.091
All-cause mortality	5/64 (7.8%)	9/51 (17.6%)	9.8 (–0.8–20.4)	2.12 (0.69–6.55)	.187

HF = heart failure, HR = hazard ratio.

* n/N (%) indicates the number of patients who experienced the outcome event within each group divided by the total number of patients in that group, expressed as a percentage. Adjusted hazard ratios (HRs) were derived from Cox proportional hazards regression models, with verification of the proportional hazards assumption.

### 3.5. Early intolerance and adverse events

The incidence of early intolerance within 30 days and specific adverse events is shown in Table 5. The frail group had a significantly higher incidence of any intolerance event within 30 days compared with the non-frail group (23.5% vs 9.4%, adjusted OR = 2.89, 95% CI: 1.08–7.76, *P* = .034). Regarding specific adverse events, the frail group showed numerically higher rates of symptomatic hypotension/volume depletion (11.8% vs 4.7%), acute kidney injury (9.8% vs 3.1%), and genitourinary infections (7.8% vs 3.1%) compared with the non-frail group, though these differences did not reach statistical significance. Euglycemic diabetic ketoacidosis and severe hypoglycemia were rare in both groups.

**Table 5:** Thirty-day early intolerance and individual adverse events.

Outcome	Non-frail n/N (%)	Frail n/N (%)	Risk difference, % (95% CI)	Adjusted OR (95% CI)	*P* value	Nagelkerke *R*^2^
Any intolerance within 30 days	6/64 (9.4%)	12/51 (23.5%)	14.1 (1.2–27.0)	2.89 (1.08–7.76)	0.034	.203
Symptomatic hypotension/volume depletion	3/64 (4.7%)	6/51 (11.8%)	7.1 (−2.4 to 16.6)	2.69 (0.61–11.87)	0.191	.098
AKI (KDIGO)	2/64 (3.1%)	5/51 (9.8%)	6.7 (−1.7 to 15.1)	3.05 (0.57–16.22)	0.189	.152
Genitourinary infection (fungal/UTI)	2/64 (3.1%)	4/51 (7.8%)	4.7 (−3.1 to 12.5)	2.60 (0.46–14.62)	0.28	.076
Hyponatremia	1/64 (1.6%)	2/51 (3.9%)	2.3 (−3.2 to 7.8)	2.33 (0.20–26.81)	0.492	.042
Euglycemic DKA	0/64 (0%)	1/51 (2.0%)	2.0 (−2.0 to 6.0)	–	–	
Severe hypoglycemia	0/64 (0%)	1/51 (2.0%)	2.0 (−2.0 to 6.0)	–	–	

AKI = acute kidney injury, CI = confidence interval, DKA = diabetic ketoacidosis, KDIGO = Kidney Disease: Improving Global Outcomes, OR = odds ratio, UTI = urinary tract infection.

* n/N (%) indicates the number of patients who experienced the event within each group, divided by the total number of patients in that group, expressed as a percentage. Nagelkerke *R*^2^ for the multivariable logistic regression models ranged from 0.076 to 0.203 across outcomes.

### 3.6. Subgroup analysis

The results of the subgroup analysis are shown in Table 6. Interaction testing indicated that baseline renal function (eGFR) was a significant effect modifier (interaction *P* = .041). Among patients with eGFR < 45 mL/min/1.73 m^2^, the association between frailty and SGLT2i intolerance was strongest (adjusted OR = 3.12, 95% CI: 1.01–9.65). In the subgroups with lower baseline systolic blood pressure (SBP < 110 mm Hg) and those receiving high-dose loop diuretics, a trend toward a stronger association between frailty and intolerance was also observed, though the interactions did not reach statistical significance. The association between frailty and intolerance remained consistent across different HF phenotypes (HFrEF vs HFmrEF/HFpEF) and in patients with or without diabetes.

**Table 6:** Subgroup effects and interaction tests.

Subgroup	Events non-frail n/N (%)	Events frail n/N (%)	Adjusted OR (95% CI)	Interaction *P*
eGFR < 45	5/22 (22.7%)	11/24 (45.8%)	**3.12 (1.01–9.65**)	.041
eGFR ≥ 45	7/42 (16.7%)	9/27 (33.3%)	2.04 (0.71–5.86)	
SBP < 110 mm Hg	4/18 (22.2%)	8/15 (53.3%)	3.78 (0.95–15.07)	.087
SBP ≥ 110 mm Hg	8/46 (17.4%)	12/36 (33.3%)	2.28 (0.82–6.34)	
Loop dose high	5/20 (25.0%)	10/19 (52.6%)	3.25 (0.95–11.08)	.122
Loop dose low	7/44 (15.9%)	10/32 (31.3%)	2.33 (0.80–6.76)	
HFrEF (LVEF < 40%)	6/28 (21.4%)	9/21 (42.9%)	2.71 (0.83–8.83)	.311
HFmrEF/HFpEF	6/36 (16.7%)	11/30 (36.7%)	2.91 (0.94–9.00)	
With DM	5/28 (17.9%)	9/27 (33.3%)	2.23 (0.70–7.08)	.524
Without DM	7/36 (19.4%)	11/24 (45.8%)	**3.52 (1.09–11.32**)	

BMI = body mass index, CI = confidence interval, eGFR = estimated glomerular filtration rate, HFmrEF = heart failure with mildly reduced ejection fraction, HFpEF = heart failure with preserved ejection fraction, HFrEF = heart failure with reduced ejection fraction, NYHA = New York Heart Association, OR = odds ratio, SBP = systolic blood pressure.

* Adjusted ORs were calculated using multivariable logistic regression, with adjustment for age, BMI, eGFR, NYHA class, loop diuretic dose, and chronic kidney disease. Interaction *P* values were calculated using likelihood ratio tests. Bolded values indicate adjusted ORs with *P* < .05.

## 4. Discussion

This study demonstrated that frailty is closely associated with the tolerability of SGLT2 inhibitors in patients with heart failure aged ≥75 years, with significant differences observed across multiple outcomes. First, regarding the primary composite endpoint, the 6-month intolerance rate was significantly higher in the frail group compared with the non-frail group (39.2% vs 18.8%), and frailty remained an independent risk factor after multivariable adjustment. This finding suggests that frailty, as a syndromic condition, may reduce the ability of elderly patients to tolerate novel HF therapies through mechanisms such as cumulative physiological decline, malnutrition, and polypharmacy.^[14]^ Clinically, in addition to routine monitoring of blood pressure and renal function, frailty screening should be considered an important tool when initiating SGLT2 inhibitors in older HF patients, to identify high-risk individuals for intolerance in advance.^[15]^

For secondary outcomes, this study found that the risk of intolerance within 30 days was significantly higher in the frail group (23.5% vs 9.4%). Early intolerance may be closely related to the initial hemodynamic effects of SGLT2i, particularly their natriuretic, diuretic, and blood pressure–lowering actions, which can more easily induce symptomatic hypotension or volume depletion in patients with reduced physiological reserve.^[16]^ At the same time, patients with unstable renal function are more likely to develop acute kidney injury (AKI) in the early phase. These results highlight that the early treatment period is a critical window for monitoring and intervention, and close follow-up during the first 1 to 4 weeks after initiation is recommended to allow timely treatment adjustments.

Regarding the spectrum of adverse events, the frail group exhibited a higher incidence of hypotension/volume depletion, AKI, and genitourinary infections, although the differences did not reach statistical significance due to the limited sample size. Mechanistically, SGLT2i may increase the risk of volume depletion through their diuretic effects, while frail patients – with impaired blood pressure regulation and reduced glomerular filtration reserve – are more vulnerable to AKI.^[17,18]^ In addition, frailty is often accompanied by impaired immune function and limited self-care capacity, which may explain the higher risk of genitourinary infections.^[18]^ Although no significant increase in severe hypoglycemia or ketoacidosis was observed, vigilance should still be maintained in clinical practice.

In the subgroup analysis, the association between frailty and intolerance was most pronounced in patients with eGFR < 45 mL/min/1.73m^2^ (interaction *P* = .041), suggesting that impaired renal function may amplify the adverse impact of frailty. In contrast, although trends were also observed in patients with lower baseline blood pressure or those receiving high-dose loop diuretics, the differences did not reach statistical significance. This may indicate that the interaction between frailty and renal dysfunction is particularly important in the elderly population, whereas blood pressure and diuretic dose may act more as auxiliary “susceptibility” factors.^[19,20]^ Clinically, in elderly frail patients with concomitant renal impairment, greater caution is warranted when initiating SGLT2i therapy, and it may even be appropriate to delay initiation or intensify monitoring.

With respect to clinical prognosis, this study found that the frail group had higher rates of HF rehospitalization, all-cause rehospitalization, and all-cause mortality compared with the non-frail group, although the differences did not reach statistical significance. Possible explanations include the relatively small sample size, short follow-up period, and limited number of events. Nonetheless, the numerical differences suggest that frailty may not only affect drug tolerability but may also indicate worse long-term outcomes. Larger-scale studies with longer follow-up are needed to further validate these findings. If confirmed, frailty screening could serve as a powerful tool for assessing prognosis in elderly HF patients.

Based on these findings, several practical considerations may help guide clinical practice. First, frailty screening using validated tools could be incorporated into pre-prescription assessment to identify patients at higher risk of SGLT2i intolerance. Second, for frail patients, particularly those with eGFR < 45 mL/min/1.73m^2^, closer monitoring of blood pressure, volume status, and renal function during the first 30 days of therapy is recommended. Third, a “start low, go slow” approach with careful dose titration may improve tolerability while maintaining therapeutic benefits. These recommendations underscore the importance of individualized treatment strategies in this vulnerable population.

Several limitations of this study should also be acknowledged. First, the sample size was relatively small, and some findings only showed trends, limiting statistical power. Second, this was a single-center retrospective study, with potential risks of selection bias and information bias. Specifically, the inclusion criterion requiring patients to be able to complete the fried frailty phenotype (FFP) assessment during a clinically stable period may have systematically excluded the most severely ill or unstable patients, thereby limiting the generalizability of our findings to the broader elderly heart failure population. Third, frailty status was assessed only at baseline and could not reflect dynamic changes in frailty over time and their impact on outcomes. Fourth, due to the absence of precise time-to-event data for SGLT2i intolerance events (e.g., exact dates of discontinuation or dose reduction), we used logistic regression and reported odds ratios (ORs) for the primary outcome, whereas hazard ratios (HRs) were used for clinical outcomes with available event times. Although this approach was justified by data availability, the use of ORs for the primary outcome precludes the consideration of time-to-event and limits causal interpretation. Furthermore, we acknowledge that mixing OR- and HR-based interpretations across different outcomes may pose challenges for readers; however, we have consistently applied each method according to the nature of the outcome and explicitly justified the rationale in Section 2 to maintain transparency. Finally, patient-reported outcomes such as quality of life were not included, which may have underestimated the overall effect of frailty on tolerability.

In summary, this study suggests that frailty may be associated with SGLT2i intolerance in HF patients aged ≥75 years, with a potentially elevated risk observed in those with impaired renal function. Although frail patients did not show statistically significant differences in hard outcomes, the observed trends warrant further investigation. For elderly frail HF patients, individualized assessment and close follow-up may be considered to balance the efficacy and safety of SGLT2i therapy. Given the exploratory nature of this single-center study with a modest sample size, these findings should be interpreted with caution. Future multicenter, large-scale prospective studies are needed to further validate these findings.

## Author contributions

**Conceptualization:** Zimeng Li, Wei Deng.

**Data curation:** Zimeng Li, Wei Deng.

**Formal analysis:** Zimeng Li, Wei Deng.

**Funding acquisition:** Wei Deng.

**Investigation:** Wei Deng.

**Writing – original draft:** Zimeng Li, Wei Deng.

**Writing – review & editing:** Zimeng Li, Wei Deng.
